# BLE Signal Processing and Machine Learning for Indoor Behavior Classification

**DOI:** 10.3390/s25144496

**Published:** 2025-07-19

**Authors:** Yi-Shiun Lee, Yong-Yi Fanjiang, Chi-Huang Hung, Yung-Shiang Huang

**Affiliations:** 1 Graduate Institute of Applied Science and Engineering, Fu Jen Catholic University, New Taipei City 242062, Taiwan; 407068021@m365.fju.edu.tw (Y.-S.L.); allen-hung@ieee.org (C.-H.H.); 2 Department of Computer Science and Information Engineering, Fu Jen Catholic University, New Taipei City 242062, Taiwan; 412226274@m365.fju.edu.tw; 3 Department of Information Technology, Lee-Ming Institute of Technology, New Taipei City 243083, Taiwan

**Keywords:** BLE-based indoor positioning, machine learning for behavior analysis, privacy-preserving health monitoring, wearable IoT for remote health tracking, AI-driven fall detection, smart home healthcare

## Abstract

Smart home technology enhances the quality of life, particularly with respect to in-home care and health monitoring. While video-based methods provide accurate behavior analysis, privacy concerns drive interest in non-visual alternatives. This study proposes a Bluetooth Low Energy (BLE)-enabled indoor positioning and behavior recognition system, integrating machine learning techniques to support sustainable and privacy-preserving health monitoring. Key optimizations include: (1) a vertically mounted Data Collection Unit (DCU) for improved height positioning, (2) synchronized data collection to reduce discrepancies, (3) Kalman filtering to smooth RSSI signals, and (4) AI-based RSSI analysis for enhanced behavior recognition. Experiments in a real home environment used a smart wristband to assess BLE signal variations across different activities (standing, sitting, lying down). The results show that the proposed system reliably tracks user locations and identifies behavior patterns. This research supports elderly care, remote health monitoring, and non-invasive behavior analysis, providing a privacy-preserving solution for smart healthcare applications.

## 1. Introduction

With the rapid advancement of technology, modern life has become inseparable from technological innovations. The application of electronic devices has evolved from early standalone sensing systems to IoT (Internet of Things) data sharing and has further advanced into AI (Artificial Intelligence) services capable of interacting with users through data. The development of these technologies aims to enhance quality of life by providing safer and more convenient assistive services. In smart home applications, beyond improving the interactivity of home appliances, smart home care has become a major research focus in recent years. With the global challenges of aging populations, declining birth rates, and shortages of long-term care resources, effectively utilizing IoT technologies to assist the elderly in accident prevention, physiological monitoring, and home security management has become a critical solution to address the challenges of “aging in place”.

Although there are currently many studies and products focused on home care, some designs suffer from issues such as system complexity, excessive user difficulty, or low usage frequency. Therefore, adaptive and intuitive designs are necessary to maintain users’ continuous engagement and effectiveness [1]. Therefore, researchers have proposed various smart care technology solutions to address potential incidents and scenarios that may occur in care environments, aiming to enhance the safety and convenience of home care. The following sections will discuss monitoring methods, behavior pattern collection, and data transmission approaches, respectively.

### 1.1. Indoor Behavior Analysis Challenges

Currently, monitoring technologies in smart home care can be broadly classified into two categories: image-based monitoring and non-image-based monitoring. The primary advantage of image-based monitoring is that the subject does not need to wear sensors, making it particularly suitable for continuous behavior pattern analysis. For example, one study installed a panoramic camera in a living room to record daily activities. By applying machine learning and mathematical algorithms, the system accurately recognized four types of behaviors—standing, sitting, walking, and falling—with an accuracy rate of up to 93%, demonstrating potential applications in disease prevention and the development of smart care systems [2,3]. Additionally, some studies have deployed RGB cameras in home environments to monitor individuals with chronic illnesses, frail elderly persons, or those living alone in real time. These systems use object detection, feature extraction, and machine learning to analyze normal and abnormal states, enabling fall detection and early warnings [4].

However, image-based monitoring raises privacy concerns. Some studies have proposed using low-resolution imaging technology to reduce image clarity while maintaining accurate analysis of abnormal human behaviors, balancing privacy protection with caregiving needs [5]. This is particularly useful in public spaces such as care facilities, where human resources may be limited or nighttime supervision is lacking. Such technologies can effectively assist caregivers in monitoring elderly individuals and preventing falls. To further improve monitoring accuracy, some studies have combined image analysis with wireless physiological monitoring. One approach involves using infrared 3D Time-of-Flight (ToF) sensors, which detect the time it takes for infrared light to reflect off surfaces, allowing the system to calculate depth and distance at different positions. This system analyzes depth images to determine whether a subject has left the bed. Additionally, comparisons with non-image-based sensing technologies, such as pressure sensors and human infrared sensors, have shown that ToF sensing technology provides more accurate subject analysis while eliminating the inconvenience of wearing sensors [6].

In smart care applications, image-based monitoring combined with deep learning technology can quickly and accurately identify behaviors that require attention. However, regardless of the resolution of imaging devices, they are not suitable for certain private spaces such as dressing rooms and bathrooms. Additionally, some monitoring devices may transmit private image data to specific servers, posing a risk of personal privacy breaches. As a result, non-image-based monitoring technologies have increasingly become the preferred choice for smart home care applications.

Non-image-based monitoring technologies primarily rely on physiological data recording and activity tracking to monitor user status. For instance, a study proposed a smart home healthcare system based on physiological monitoring, where wearable devices measure biometric signals and record data to analyze long-term trends and predict disease risks, enabling early intervention. When specific physiological indicators, such as ECGs (Electrocardiograms), exhibit abnormalities, the system can immediately notify the user, family members, or medical personnel to provide real-time alerts and response measures [7,8].

In non-image-based monitoring, wearable device data transmission depends on wireless communication technologies to transfer sensor data to smart devices or servers for analysis and storage. Common wireless transmission technologies include ZigBee, BLE (Bluetooth Low Energy), Wi-Fi, and UWB (Ultra-Wideband). However, since IoT systems are generally designed with cost-effectiveness and open-source principles in mind, communication methods between devices must be widely compatible. Many household devices and smart appliances are already equipped with BLE or Wi-Fi modules. Considering the need for stable long-distance transmission and direct Internet connectivity, some studies have proposed using Wi-Fi communication to enable remote access and control of devices [9].

### 1.2. BLE-Based Non-Visual Monitoring Needs

Wireless communication technologies not only enable data transmission but also allow for distance estimation by measuring the time taken during data transmission. This capability is useful for indoor positioning and behavior analysis. For instance, studies have explored the feasibility of using UWB for indoor positioning due to its advantages of wide frequency spectrum, low power consumption, and minimal interference [10]. However, the cost of UWB-based positioning devices is relatively high, and the monitored individuals must wear specific devices for the system to function.

Common positioning technologies include GPS (Global Positioning System) and GSM (Global System for Mobile Communications). While GPS is highly effective for outdoor positioning, it does not provide accurate indoor location information. GSM can complement GPS in certain environments, but its indoor positioning accuracy remains limited. As an alternative, some studies have proposed an indoor positioning method based on RSSI (Received Signal Strength Indication) trilateration, utilizing the wireless transmission process of smart devices [11].

Modern smart devices are typically equipped with wireless communication modules such as GSM, Wi-Fi, and Bluetooth. Among them, Wi-Fi is the most commonly used for short-range data transmission. Some research has explored Wi-Fi signal strength indicators combined with location fingerprinting techniques for indoor positioning [12,13]. However, Wi-Fi-based positioning requires multiple access points, leading to higher infrastructure costs and limiting widespread adoption. With advancements in hardware technology, BLE-based positioning has gained increasing attention. By comparing the RSSI values of BLE DCUs, researchers have developed cost-effective and easy-to-deploy indoor positioning solutions. Compared with Wi-Fi-based systems, BLE positioning offers lower costs and greater deployment flexibility. Consequently, many studies have proposed BLE-based indoor positioning and behavior analysis techniques, where multiple BLE DCUs are installed at different locations, and RSSI values are used to determine user positions and track movement patterns [14,15], providing an effective solution for smart care applications.

### 1.3. Data Transmission Methods Suitable for Small-Scale IoT Systems

Many IoT devices face processing limitations due to constraints in computational power and network transmission resources, making it necessary to minimize the data size of each transmission. A common method for data uploading and storage is by using HTTP (Hyper Text Transfer Protocol). In addition to HTTP services, MQTT (Message Queuing Telemetry Transport) is a lightweight communication protocol designed for low-bandwidth, high-latency, or unstable network environments. It is particularly suitable for low-power devices such as sensors and smart wristbands and is widely used in IoT device communications [16,17]. MQTT employs a Publish/Subscribe mechanism, allowing each device to function as a client that communicates with a broker server. This architecture provides a more efficient, low-latency data transmission method compared with traditional request–response protocols, making it an ideal choice for real-time IoT applications.

**Figure 1:**
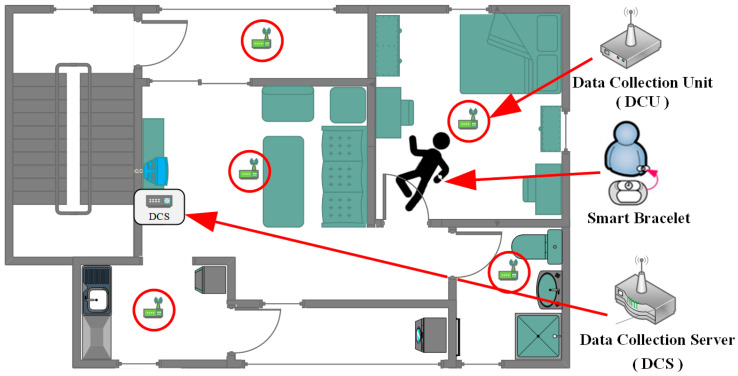
System overview.

The intelligent home care system designed in this study aims to address several challenges currently faced in elderly care and home safety. It introduces a system that analyzes signal strength data from BLE smartwatches for behavior recognition, as illustrated in Figure 1. Based on issues identified during previous research, the following improvements have been proposed to enhance DCU positioning accuracy, data consistency, and signal processing stability.

#### 1.3.1. Adding Vertical Wall-Mounted DCUs for Height Positioning Optimization

In a previous study, all DCU devices were installed on the ceiling. After performing triangulation, the coordinates were converted into distances between the triangulated point and each DCU to calculate the adjacent sides in the Pythagorean theorem. Then, the RSSI values were converted into distance values to determine the hypotenuse, which was subsequently used to calculate the opposite side. However, when the DCU devices were affected by external environmental interference, the hypotenuse calculated from the RSSI values would be inaccurate, thereby impacting the final height estimation.

To address this issue, the current study proposes adding a DCU device on a vertical wall and integrating it with two ceiling-mounted DCUs. By utilizing the vertical angle of indoor space for positioning, this setup provides more accurate user height data and mitigates errors caused by using incorrect RSSI-derived values for height estimation.

#### 1.3.2. Synchronizing DCU Data Collection Time to Improve Data Consistency

Data from the previous study revealed that the original data collection method often led to significant imbalances, with some DCUs collecting more than twice the amount of data compared with others. To address this, the current study proposes an optimized data collection mechanism for DCU devices. This new approach ensures that all DCUs record data simultaneously at the same time points, thereby reducing instances where certain DCUs receive significantly more or less data. This synchronization improves data consistency, which is essential for subsequent data processing and machine learning training.

#### 1.3.3. Applying Data Smoothing Techniques to Reduce Environmental Interference

In the previous study, data processing was conducted using the Mean Filter and Median Value Average Filter. However, both filtering methods were highly susceptible to environmental interference, often resulting in significant discrepancies between the calculated results and actual conditions. Therefore, in this study, the RSSI signal data is preprocessed using either a Kalman Filter or a Moving Average Filter. These methods help smooth the RSSI signals and reduce anomalies caused by environmental noise, thereby enhancing the stability and accuracy of subsequent data analysis.

#### 1.3.4. Integrating AI for RSSI Data Analysis to Enhance Behavior Recognition

Machine Learning (ML) and Large Language Models (LLMs) are utilized to analyze BLE RSSI data. AI-based pattern recognition is employed to identify user behaviors or analyze RSSI variation trends under different behavioral patterns, improving the system’s ability to recognize and classify behaviors.

## 2. System Architecture

### 2.1. Advantages of Proposed System

**Figure 2:**
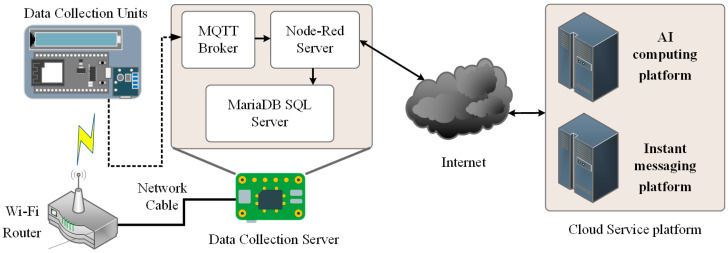
System architecture diagram.

To avoid potential privacy concerns associated with using camera-based monitoring of test subjects, this study builds upon the previously proposed concept, “Design and Implementation of an Indoor Alert System for Physiological Signal Monitoring of Home-Isolated Individuals” [18]. It adopts Bluetooth Low Energy (BLE) technology to analyze the Received Signal Strength Indicator (RSSI) between signal-emitting devices and smart devices. Indoor positioning and behavior analysis are conducted on edge devices and cloud platforms, respectively, enabling the development of a non-visual, intelligent home care monitoring system. The system architecture is illustrated in the following Figure 2.

This study further optimizes the system architecture and data collection mechanism by equipping test subjects with a smart wristband and deploying multiple DCU devices within a designated home environment, along with a data collection and analysis system. Additionally, Machine Learning is integrated to enhance data analysis capabilities, further improving positioning accuracy and behavior recognition precision. This enables the implementation of a more stable and efficient non-intrusive monitoring mechanism.

### 2.2. Hardware Architecture

#### 2.2.1. Data Collection Unit (DCU)

**Figure 3:**
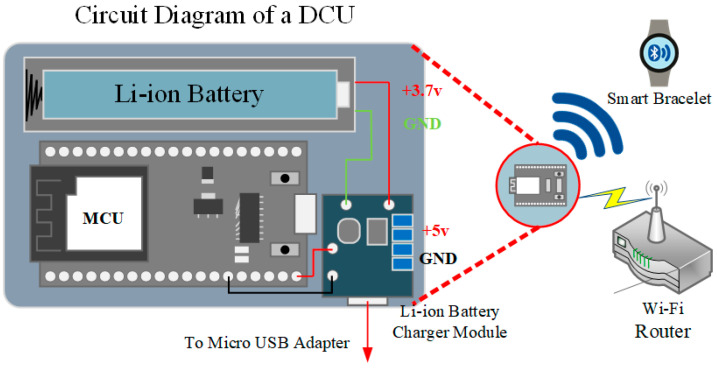
Data collector hardware architecture.

The data collector comprises a microcontroller, a Bluetooth module, a wireless network module, and a lithium battery charging module. This study employs the ESP32 chip (ESP32-WROOM-32 module manufactured by Espressif Systems, Shanghai, China) as the microcontroller, leveraging its built-in Bluetooth Low Energy (BLE) and Wi-Fi capabilities. The integrated module architecture reduces the need for additional external components, lowering costs and minimizing the overall device size [19]. The hardware architecture is illustrated in Figure 3.

In this study, the ESP32 microcontroller functions as the DCU device for the data collector, responsible for detecting and scanning BLE signals from nearby smart wristbands, recording RSSI signal strength, and transmitting the data to a server via Wi-Fi for further analysis. Each DCU is equipped with a lithium battery and a charging circuit. If an external power adapter fails, the system can seamlessly switch to lithium battery power, ensuring uninterrupted device operation and enhancing system stability and reliability.

To improve the accuracy of BLE signal collection and enhance the transparency of system design, this study further supplements the relevant parameters of the BLE devices used, as well as the environmental factors affecting behavior recognition accuracy, as detailed below:

1. Sampling Rate:
  This study used the ESP32 module as the BLE scanning device, with the scan interval set to 500 ms, meaning that RSSI signals were collected twice per second. This sampling rate, as verified through experiments, is sufficient for detecting changes in user behavior during static and general activities (such as lying down, sitting, and standing) while avoiding excessive computational and communication burdens on the system.
2. Detectable Range:
  In an indoor open-space environment, the ESP32 module’s detectable BLE signal range is approximately 10 to 20 m. However, the actual effective range can be influenced by environmental factors (such as walls, furniture, and human movement). In this study’s experimental setting— a typical home living room—the average distance between the DCU and wristband devices was maintained within 5 m, ensuring stable RSSI signal collection.
3. Wall Penetration Capability:
  Since BLE operates in the 2.4 GHz frequency band, its signal undergoes noticeable attenuation when passing through obstacles like walls. Nevertheless, basic communication capabilities can still be maintained through common building materials (such as wooden boards and lightweight walls). This study’s experiment was primarily conducted in a single open area (the living room) without addressing multi-room recognition. If the system is expanded to cover the entire home in the future, further evaluation will be conducted on how various wall materials affect RSSI attenuation, and corresponding strategies will be incorporated into environmental modeling and algorithm adjustments.

#### 2.2.2. Smart Wristband

The smart wristband is primarily used to identify the user’s location by periodically broadcasting BLE signals (including the MAC address, device ID information, etc.), allowing BLE DCU devices to detect and record the user’s location data. Each smart wristband has a unique MAC address (Media Access Control Address), which serves as a means to distinguish between different users. The system can effectively differentiate individuals, perform precise indoor positioning, conduct behavior analysis, and track data trends by having test subjects wear these wristbands.

This study uses commercially available smartwatches equipped with BLE and physiological signal measurement capabilities as the smart wristbands for collecting user data. Commercial smartwatches are chosen because they more closely resemble everyday accessories worn by most people, eliminating the need to design specialized wearable devices that may not meet user needs or are only suitable for specific scenarios. Preference is given to smartwatches that can synchronize with the Google Health Connect service, enabling more accurate assessment of physiological states in the future through synchronized physiological measurement data.

This study tested the Xiaomi Smart Band, Amazfit Pop, and Samsung Watch. All three smartwatches have apps that support synchronization with Google Health Connect and BLE broadcasting. During testing, the Xiaomi Smart Band 5 and Amazfit Pop were able to continuously broadcast BLE signals. However, due to software design aimed at extending battery life, the Samsung Watch stops broadcasting after a period if no pairing device is found, requiring repeated manual activation of BLE broadcasting to complete RSSI measurements.

The Xiaomi Smart Band, limited by its smaller size and 125 mAh battery capacity (compared with the Amazfit Pop’s 225 mAh), has a shorter measurement duration. Additionally, when the battery level drops below 10%, the RSSI signal strength of BLE broadcasting decreases. Therefore, this study uses the Amazfit Pop as the primary device for user identification during the measurement process.

#### 2.2.3. Data Collection Server (DCS)

This study utilizes an embedded computing device, Raspberry Pi 4 Model B (Raspberry Pi Foundation, Cambridge, UK), as the Data Collection Server. Compared with traditional computer server architectures, Raspberry Pi offers low power consumption, cost-effectiveness, and a compact form factor, making it well suited for smart home healthcare applications. Additionally, the system is integrated with a GSM module, addressing connectivity issues in environments where WLAN is unavailable [20].

**Figure 4:**
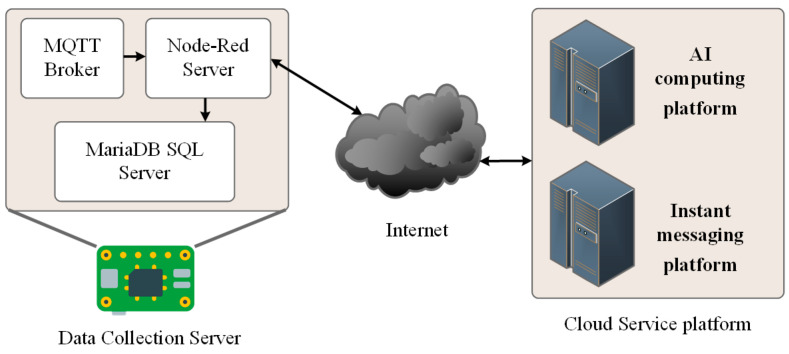
Data collection server software architecture.

Figure 4 illustrates the system’s operational workflow. The system is designed to operate in a local processing mode, where all data is stored on the embedded computing device. For computational tasks requiring a GPU (Graphics Processing Unit), such as Machine Learning or LLM analysis, data is first preprocessed locally before being transmitted via the Internet to a cloud server for further analysis.

### 2.3. Data Collection Server Architecture

#### 2.3.1. Data Transmission

As this study adopts a non-visual monitoring approach, test subjects are required to wear sensors or signal transmitters, with data being transmitted wirelessly. However, the complexity of IoT environments introduces several challenges, including hardware limitations, network stability, bandwidth constraints, power consumption, and the number of connected devices, all of which impact data transmission efficiency and reliability. For IoT data transmission, two widely used protocols with extensive support are Web API (Web Application Programming Interface) and MQTT (Message Queuing Telemetry Transport). This study selects MQTT due to its advantages, including low data transmission overhead, stable communication, and efficient monitoring capabilities. Therefore, MQTT is employed as the primary data transmission protocol between the DCU devices and the DCS.

#### 2.3.2. Data Management

The system employs Node-Red as the primary tool for data and message management. Node-Red is a visual development platform built on Node.js, specifically designed for IoT applications. It provides a flow-based visual programming environment, enabling users to develop IoT solutions via a browser-based interface. Additionally, custom nodes can be installed to extend system functionalities and services [21,22].

In this system, an MQTT broker and a database are installed within Node-Red. Through specific node functions, it manages all data uploaded from the DCUs and categorizes and stores the data in the database according to predefined procedures. Upon receiving RSSI data from each receiver node, an external Python v3.11.2 processing script is immediately triggered. This script performs preliminary edge computing tasks, including time synchronization, data gap filling, signal smoothing techniques such as Kalman filtering or moving average, and sliding window segmentation and format conversion. Through these preprocessing steps, the raw and highly volatile RSSI signals are transformed into vector data suitable for backend model analysis.

#### 2.3.3. Data Storage

Common data storage solutions can be categorized into cloud databases and local databases. Cloud databases offer stable and secure data storage services, whereas local databases are limited by the memory capacity and lifespan of hardware devices. Since this study primarily analyzes the behavior patterns of individuals living alone, large-scale data transmission may be unfeasible in certain environments due to network constraints. Therefore, a local database is deployed within the DCS to ensure reliable data management. The system utilizes MariaDB as the database management system. MariaDB is an open-source alternative to MySQL, offering enhanced performance and additional features not available in MySQL. It is compatible with most MySQL-based applications and converters, making it a suitable choice for the system. Additionally, MariaDB is one of the primary database solutions supported by Raspberry Pi, ensuring seamless integration with the hardware infrastructure.

### 2.4. Research Methodology

#### 2.4.1. Data Measurement Method

**Figure 5:**
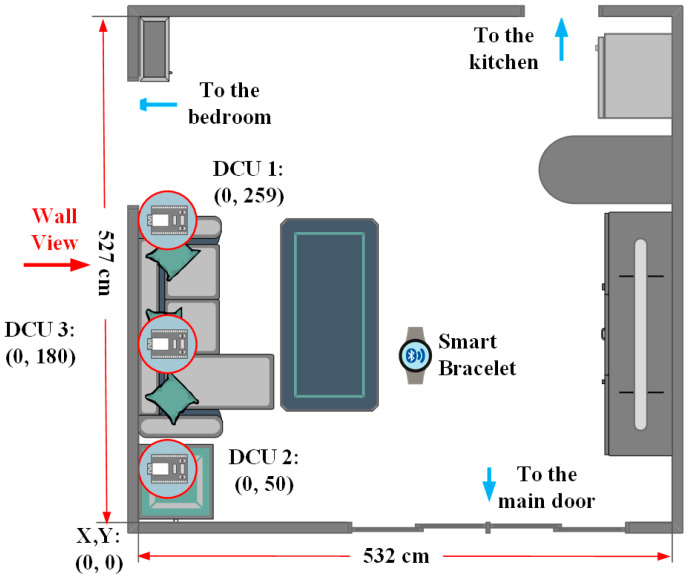
A prototype testing environment.

Figure 5 illustrates the indoor layout of the test environment, which covers an area of approximately 27.8 m^2^. This prototype environment represents the researcher’s actual living space, where a DCU is installed on one of the walls. To establish a spatial coordinate system, the bottom-left corner of the room is defined as the origin (0, 0). The actual room dimensions are set to 532 cm × 527 cm based on real-world measurements. Three DCUs are installed on the left-side wall of the living room, positioned as follows:

1. DCU 1 (259, 250):
  Mounted above the entrance connecting the living room to the bedroom.
2. DCU 2 (50, 250):
  Installed above a tabletop, near the bottom-left corner.
3. DCU 3 (180, 95):
  Serves as a vertical reference point for triangulation-based positioning.

These three DCUs continuously receive RSSI data from nearby BLE devices and transmit the collected data to the DCS for storage and analysis. Additionally, to simulate the subject’s location, a smart wristband is placed at the center of the test environment. Various behavioral scenarios are designed to collect environmental data for further analysis.

**Figure 6:**
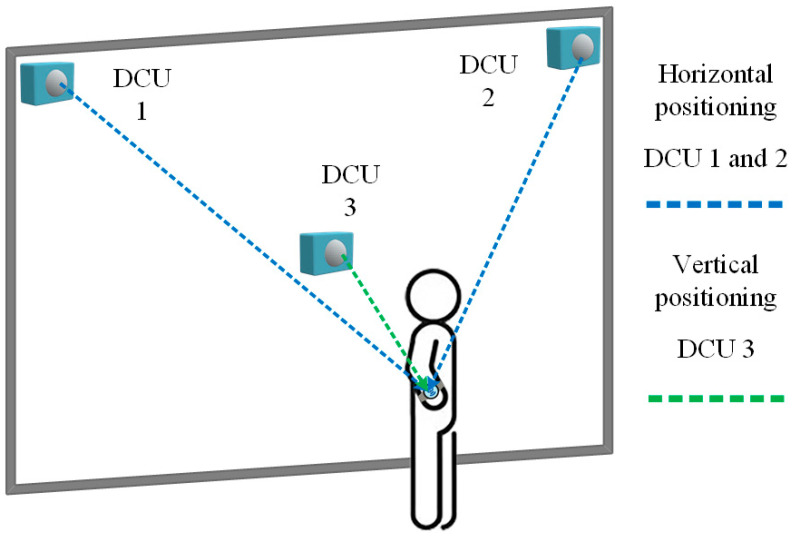
Vertical angle positioning schematic diagram.

To ensure stability in BLE data collection, this study selected a wall with minimal environmental interference for the installation of three DCU devices. Two DCUs are mounted near the ceiling, responsible for horizontal positioning and detection. Another DCU is installed on the wall, serving as a vertical positioning reference point. Additionally, the ceiling-mounted DCUs can operate in coordination with other DCUs at the same height, further enhancing horizontal positioning accuracy. This setup enables more precise spatial analysis of the user’s position within the environment, as shown in Figure 6.

**Figure 7:**
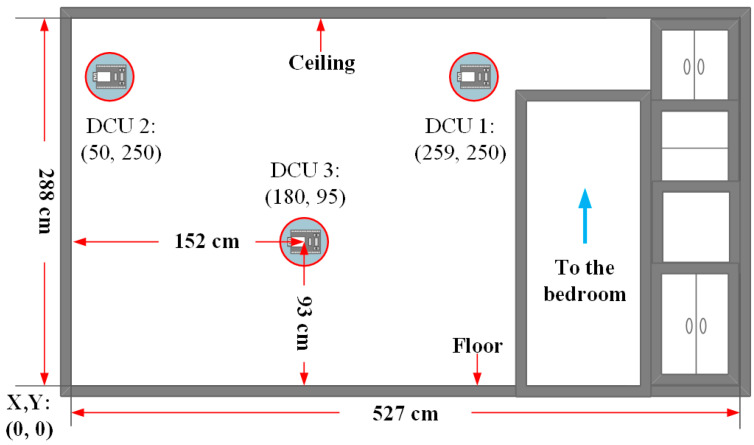
DCU device deployment diagram.

After testing the installation positions of the DCU devices, it was found that placing them in corners tends to cause a dish antenna effect. This phenomenon occurs because signals reflect off surfaces such as walls and ceilings, increasing the number of reflections. As a result, the receiving end may detect multiple signal paths, leading to constructive or destructive interference. This interference affects signal interpretation, causing the receiver to misidentify the signal source direction, ultimately increasing distance estimation errors. Therefore, when installing DCU devices, they should not be placed in corners and must be kept at a distance of approximately 50 cm from walls or protruding interior structures to minimize signal interference caused by reflections. As shown in Figure 7, the DCU installation method proposed in Figure 6 was applied in the living room environment depicted in Figure 5. The devices were installed on the wall marked with the “Well View” perspective for testing.

#### 2.4.2. RSSI Data Collection Method

This study utilizes the ESP32 microcontroller as the DCU device for collecting BLE signals. The ESP32’s built-in antenna (PCB trace antenna), under its default output setting (typically 0 dBm), can achieve a maximum range of approximately 30 m in unobstructed conditions. However, in indoor environments, signal stability is often limited to a range of 5–10 m due to obstructions such as walls, furniture, and human bodies.

In Taiwan, the typical size of a household living room is generally under 30 m^2^, which falls within the ESP32’s allowable range for BLE signal measurement in indoor settings. To ensure the DCU device can effectively scan for the smartwatch, this study first conducted a signal attenuation test between the two devices. The test began at a distance of 100 cm and increased in 100 cm increments up to 1000 cm. At each measurement point, RSSI data was collected for 10 min.

**Figure 8:**
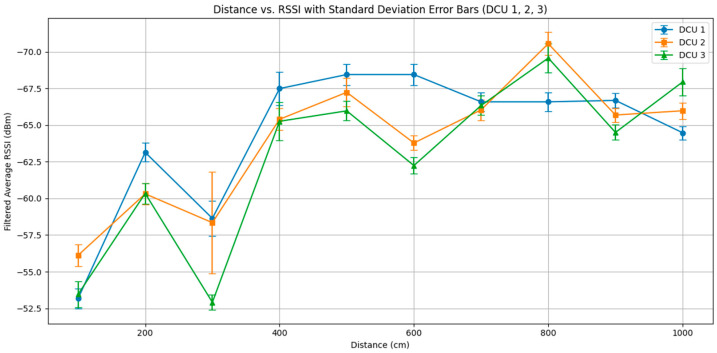
Distance vs. RSSI with standard deviation error bars.

Figure 8 illustrates the average RSSI performance of three devices (DCU 1, DCU 2, and DCU 3) at various distances, along with standard deviation error bars for each distance point to reflect signal stability. The x-axis represents the distance between the device and the signal source, while the y-axis shows the RSSI values (in dBm) after per-minute averaging and Kalman filtering. A lower RSSI value indicates a weaker signal.

The overall trend observed across the three DCUs aligns with the basic physical principle that “the greater the distance, the weaker the RSSI,” reflecting radio signal attenuation over distance. However, noticeable downward fluctuations in the RSSI curves appear at the 300 cm, 600 cm, and 900 cm points—significantly lower than neighboring points. These anomalies may be attributed to specific interferences such as multipath reflections, obstruction effects, or signal attenuation due to antenna orientation.

The error bars in the figure show the variability of RSSI at each distance. Longer bars indicate greater signal fluctuation and lower stability at that distance, whereas shorter bars suggest a more stable signal. Overall, while RSSI tends to decrease steadily over medium-to-long distances, the high variability and standard deviations at some points highlight the importance of accounting for environmental factors when performing wireless positioning or signal strength analysis.

Additionally, the results confirm that using ESP32 to scan BLE signals is reasonably stable within a 1000 cm range. Since distances beyond 1000 cm exceed the dimensions of the living room environment used in this study, such measurements are excluded from the analysis scope.

**Figure 9:**
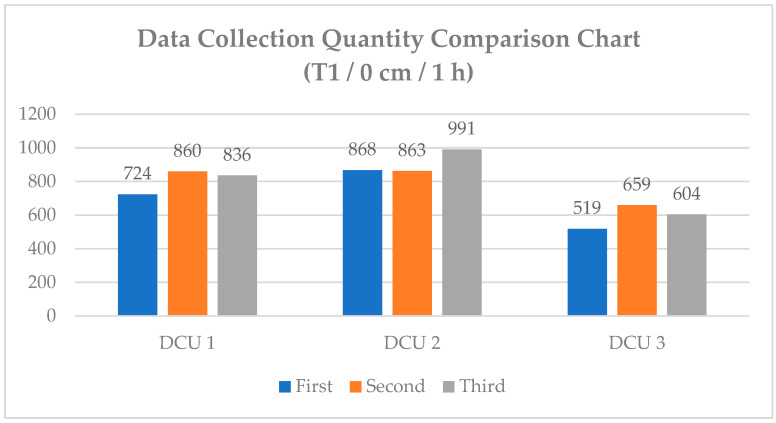
Comparison of data collection volume at position T01 (0 cm/1 h).

To analyze the user’s relative height, all three DCU devices must synchronously collect the RSSI signals transmitted by the smart wristband [23]. In previous research settings, each DCU would immediately upload the received RSSI signal to the DCS upon detecting a BLE device. However, due to environmental interference and other factors, RSSI signal collection times could vary, leading to inconsistent time intervals between DCUs. This inconsistency results in a discrepancy in the amount of data collected across different devices. As shown in Figure 9, even when data collection starts at the same initial time, the three DCUs exhibit significant differences in collected data volume, with some showing large discrepancies. If the data volume of a particular DCU device is significantly higher than that of other DCU devices, the decision tree tends to favor learning from this type of data during training. As a result, the model performs well in predicting data from the device with the largest data volume but performs poorly for DCU devices with lower data volumes. This phenomenon may affect subsequent filtering processes and reduce the accuracy of the machine learning model [24].

The primary cause of this situation lies in the design of the BLE scanning functionality using the BLEScan class library compatible with ESP32. In this library, the default scan interval (setInterval) is set to 100 ms, and the scan window (setWindow) is set to 99 ms, which theoretically allows up to 10 scans per second. However, the actual number of scans can be reduced due to factors such as the broadcast interval of the smartwatch, the execution flow of the system design, and external environmental interference. In the current system workflow, once BLE scanning is initiated, the ESP32 performs up to approximately 20 scans within a 2 s period. During this process, it collects data only from devices with the specified BLE MAC address. If multiple data packets from the same device are detected within the 2 s window, only the most recent data is sent to the DCS (Data Collection Server), followed by a 1 s wait before the next scan cycle.

During the BLE signal reception process, environmental noise interference may cause the DCU device to enter an infinite loop, where the scanning process does not stop until the system crashes and restarts. This can lead to unexpected device failures and interruptions in data collection. To address the issue of potential software crashes in the DCU under certain conditions, this study incorporates an emergency stop command into the BLE signal detection process to prevent the device from entering an infinite loop. When the DCS receives a signal from the Microwave Motion Sensor Module, indicating that a person has entered a designated area, it sends an MQTT message to all DCU devices to initiate BLE signal detection. During the detection process, after each BLE scan activation, the system waits 5 s before sending the next detection command. Once all DCU devices successfully return RSSI data, the DCS sends another detection notification and resets the 5 s countdown timer for the next scanning cycle. This implementation ensures synchronized data collection, prevents system failures, and enhances the reliability of indoor positioning and behavior analysis.

If, after 5 s of detection activation, any of the three DCU devices fails to return data, the system will send a second detection notification. If, after the second detection notification, the system still does not receive responses from all DCU devices, the forced termination function will be activated, and the system will proceed to the next detection cycle.

**Figure 10:**
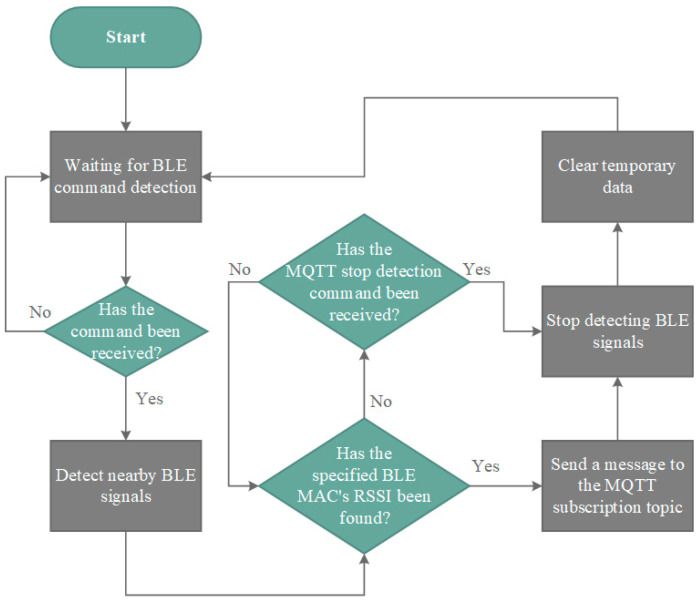
ESP32 software control flow chart.

To mitigate unexpected crashes of DCU devices, a search interruption command is integrated into the BLE detection workflow. Upon receiving an MQTT message instructing it to stop detection, the DCU device terminates all active processes, clears the temporary data storage array, and retransmits a detection notification. This mechanism ensures a proper system reset, restoring the device to a standby state where it awaits the next MQTT command to initiate BLE signal detection, as illustrated in Figure 10. The firmware was developed using Arduino core (v1.8.19) for the ESP32 (v3.2.0, Espressif Systems, Shanghai, China) in combination with the ESP32 BLE Arduino library and PubSubClient MQTT library (v2.8).

#### 2.4.3. Behavior Pattern Data Collection Method

To collect a large volume of stable data for specific postures and minimize anomalies caused by excessive environmental interference (such as human body obstruction or the difficulty of maintaining fixed actions while wearing a smartwatch), the measurement phase in this study avoids having users wear the smartwatch during data collection. Instead, alternative equipment is used to fix the smartwatch at designated reference points.

To simulate user postures in the measurement environment, smart wristbands are set up at different locations within the testing space. A telescopic selfie stick with standing functionality is used as a mounting stand to position the wristband, simulating the natural arm-hanging height when worn by a user.

The relative height thresholds for different postures are determined based on the average male height of 170 cm and are adjusted according to the typical hand positions when a user is lying on the floor, sitting on a sofa, or standing still. Considering that the accuracy of BLE signal positioning is generally around 1 to 5 m, achieving precision within 30 cm remains technically challenging with current devices and technology.

**Figure 11:**
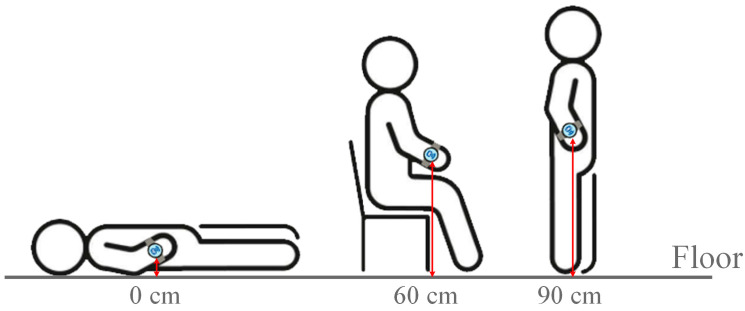
Three types of behavior.

Therefore, the baseline is set at approximately 90 cm, representing the natural arm-hanging height when a user is standing and wearing a smart wristband. When sitting or reclining on a standard sofa, the hand position typically ranges from 50 to 60 cm, while lying flat on the floor places the hand height between 0 and 15 cm. Accordingly, the posture-related height thresholds are defined as 0 cm (lying down), 60 cm (sitting), and 90 cm (standing), as illustrated in Figure 11.

## 3. Experiment Methodology

In previous research, several challenges were encountered in data collection and analysis. One major issue was that BLE data collection was highly susceptible to environmental noise interference. Additionally, the earlier setup involved installing three DCUs at the corners of the ceiling to collect data; however, when performing high-precision trilateration, the calculated results failed to effectively converge toward the central point. This led to significant deviations between the analyzed and actual positions [25]. To improve accuracy, this study integrates Machine Learning and LLMs to optimize the data processing workflow. Furthermore, solutions are proposed to address the issues identified throughout the research process.

### 3.1. Data Processing

**Figure 12:**
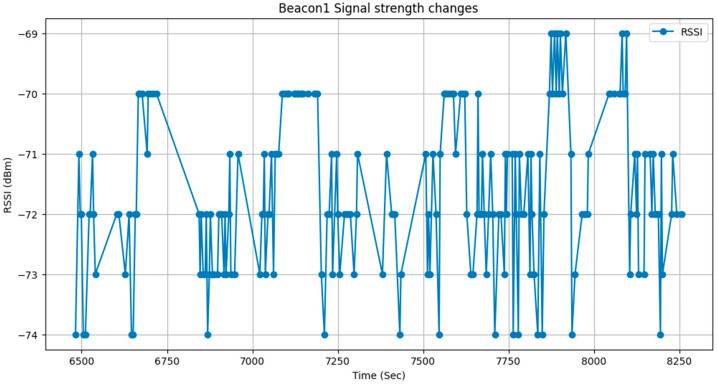
Raw data without slope filtering.

When using ESP32 as a BLE DCU device, the PCB antenna serves as the primary medium for signal reception. Due to its fixed radiation pattern, signal strength may vary depending on directional orientation. In certain directions, the signal may become weaker or be significantly affected by nearby metal objects, such as batteries, metal enclosures, and other electronic components. These factors can cause large fluctuations in RSSI signal strength, ultimately affecting data stability. Figure 12 illustrates the RSSI data trend collected by the ESP32 DCU device.

From the figure above, it can be observed that even when the test device remains at the same height without movement, the RSSI signal is still influenced by device characteristics and environmental noise. This results in signal fluctuations ranging between −69 dBm and −74 dBm, with a maximum signal variation ranging from −69 dBm to −73 dBm. Additionally, data collected from other DCU devices indicate that RSSI values can fluctuate by as much as ±6 dBm. To enhance data stability, RSSI signal smoothing is required to ensure accuracy in subsequent analysis.

Previous studies have attempted to reduce environmental interference on RSSI values using Mean Filter and Median Value Average Filter methods [26]. The Mean Filter is a linear filtering algorithm that is computationally simple and easy to implement. Its fundamental concept involves calculating the average value of RSSI measurements over a specific time window to mitigate random noise interference. This filtering method can be represented by the following mathematical model:

(1) $${\tilde{x}}_{i}=median(x_{i-k},x_{i-k+1},\ldots ,x_{i},\;\ldots ,\;x_{i+k})$$

**Figure 13:**
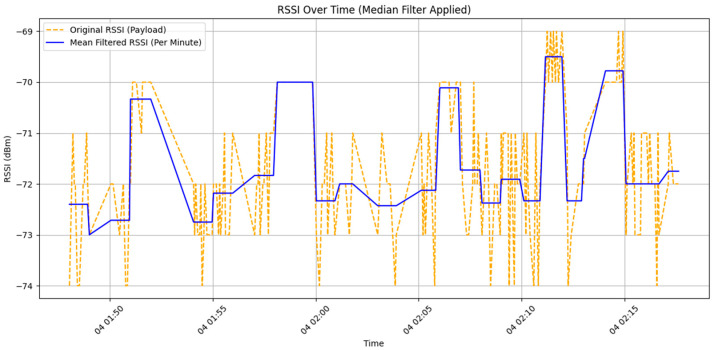
Comparison chart of raw data and mean filtered data.

Figure 13 shows the results of applying a Mean Filter to the RSSI signal data collected by DCU 1 at a height of 0 cm. During the data filtering process, a moving average is calculated using the data sampled per second to obtain a single smoothed value. As a result, the number of samples per second (*n*) is approximately 5 to 8. From the chart, it can be observed that even after filtering, a discrepancy of up to 2.5 dBm still exists.

The Median Value Average Filter combines the advantages of both the Median Filter and the Mean Value Filter. It is particularly effective in suppressing sporadic impulse noise, which helps reduce sampling deviations caused by sudden signal spikes. Its calculation method involves first grouping the data by timestamp, with each group consisting of the RSSI values collected within a one-minute interval. For each minute, all RSSI values within that timeframe are extracted, and the arithmetic mean is calculated to represent the signal value for that minute. This approach effectively smooths short-term noise fluctuations and reflects the overall signal trend.

(2) $${\tilde{x}}_{m}=\frac{1}{N}\sum_{i=1}^{N}{\tilde{x}}_{i}$$

It is especially suitable for scenarios with a sufficient number of samples per minute—typically five or more RSSI readings—to ensure stable and reliable results.

**Figure 14:**
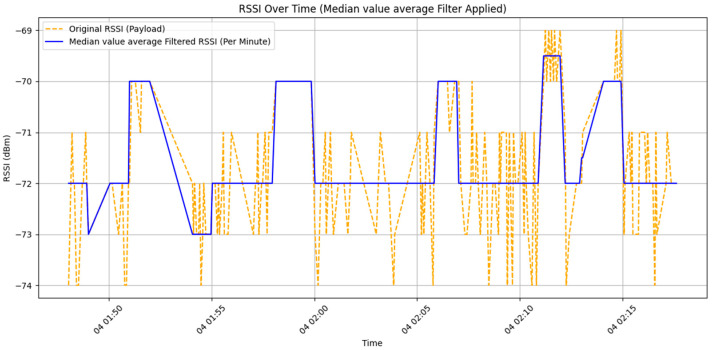
Comparison chart of raw data and median value average filtered data.

Figure 14 presents the results of applying a Median Value Average Filter to the RSSI signal data collected by DCU 1 at a height of 0 cm. The sampling method used during data filtering is the same as that of the Mean Filter, with approximately 5 to 8 samples per second (*n*). From the chart, it can be observed that while the filter reduces the jagged and unstable waveform seen in the Mean Filter process, the data still exhibits fluctuations of more than ±1.5 dBm.

In the Mean Filter data processing method, since it simply computes the average value of the collected data, it remains highly susceptible to interference. As a result, even when the device remains at the same height, signal fluctuations of up to ±7 dBm can still occur. When using the Median Value Average Filter, the impact of outlier values is reduced to some extent. However, if abnormal values are not limited to the maximum or minimum data points, significant variations in RSSI values at the same height may still persist.

In the smoothing process of RSSI data, common filtering methods include the Moving Average Filter and the Kalman Filter. Among the moving average techniques, the Exponential Moving Average (EMA) is considered more suitable. Compared with the traditional Simple Moving Average (SMA), EMA responds more quickly to changes in the data and helps reduce excessive delay, making it especially effective for real-time sensor data processing. The calculation formula is as follows:

(3) $${EMA}_{n}=\alpha \cdot x_{n}+(1-\alpha )\cdot {EMA}_{n-1}$$

**Figure 15:**
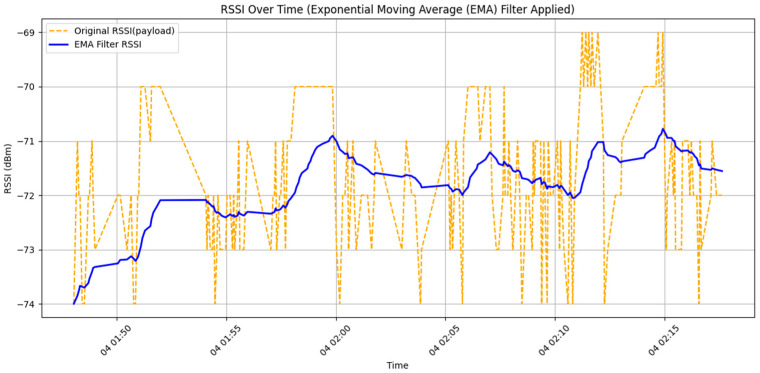
Comparison chart of raw data and exponential moving average filtered data.

Figure 15 shows the results of smoothing using the Exponential Moving Average (EMA) method. In the figure, the orange dashed line represents the raw, unprocessed RSSI values, while the blue solid line indicates the EMA filtering result with a smoothing factor α = 0.05. The parameter *α* is the smoothing coefficient, which determines the weight of the current data point in the overall filtering result.

From the figure, it can be observed that the raw data exhibits high-frequency disturbances and noticeable discontinuities, indicating a high degree of susceptibility to instantaneous interference and measurement errors. As a result, directly using such data for positioning or behavior recognition would reduce accuracy. In contrast, the EMA-filtered curve demonstrates a significantly smoother and more continuous trend, effectively suppressing noise and short-term fluctuations in the original data while preserving the overall trend in signal strength changes.

Due to the relatively small smoothing factor (α = 0.05), historical data carries greater weight in the weighted average, resulting in a more stable filtering output. This setting is particularly suitable for evaluating RSSI stability in static environments or analyzing long-term behavioral trends. However, such a low α leads to slower responsiveness when RSSI values change rapidly, which could pose a delay risk in applications that require high real-time performance, such as rapid movement detection or emergency event alerts. Therefore, when applying this filtering method in practical scenarios, the α value should be adjusted based on the dynamic characteristics and accuracy requirements of the application to achieve an optimal balance.

Compared with the EMA filter, which still exhibits a data error margin of ±2 dBm in its results, this study ultimately adopts the Kalman Filter for data smoothing. The Kalman Filter is directly applied for RSSI signal smoothing. The Kalman Filter is an optimization algorithm based on Linear Minimum Mean Square Error (LMMSE) and Linear Recursive Update. It updates the current estimated value using the previous prediction and the current measurement, effectively mitigating Gaussian noise interference. Due to its ability to handle normally distributed noise, the Kalman Filter is well suited for RSSI signal smoothing, significantly enhancing data stability and accuracy [27].

**Figure 16:**
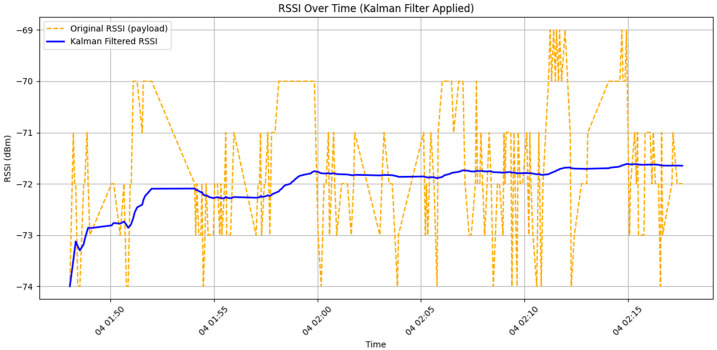
Comparison chart of raw data and Kalman-filtered data.

As shown in Figure 16, the chart displays the RSSI signal data collected by DCU 1 at a height of 0 cm, alongside the results after applying the Kalman Filter. The key parameters of the Kalman Filter include the process noise variance (*Q*) and the measurement noise variance (*R*), both of which directly influence the filter’s responsiveness and smoothing effect.

To estimate the measurement noise, this study uses the variance of the raw RSSI data as the value for *R*. Based on the collected data, the RSSI variance was calculated as:

(4) $$R=Var\left(RSSI\right)=1.6927$$

Given that system state changes over short periods are typically much smaller than measurement errors, this study follows a commonly used empirical rule found in the literature to set the process noise variance as:

(5) Q=0.01×R=0.0169

This configuration allows the Kalman Filter to effectively suppress noise while preserving the overall trend of RSSI signal variation. The filtered data remains within a more stable range, approximately around −72 dBm, with fluctuations controlled within ±1 dBm. This level of smoothing greatly benefits subsequent data analysis, enabling the system to perform positioning and decision-making with improved accuracy.

### 3.2. Machine Learning-Based Behavior Analysis

This study utilizes RSSI signal data collected by BLE Data Collection Units for behavior classification and integrates machine learning models for training and deployment. Since model training typically requires processing large datasets and performing complex computations, the training is conducted in a cloud environment. The trained model is then deployed to the DCS system to enable edge computing analysis.

The chosen model is a Decision Tree, known for its simple structure and high computational efficiency. It requires minimal data preprocessing, making it especially suitable for deployment on resource-constrained devices such as the Raspberry Pi used in this study. As a supervised learning method, the Decision Tree model is trained on labeled RSSI data to construct corresponding classification rules [28].

During the deployment phase, the trained pkl model file and preprocessing modules are uploaded to the Raspberry Pi. A complete data collection and inference workflow is established. When the system is operational, BLE data is received and processed via Node-Red. A Python script using the Flask framework then invokes the model for real-time inference, completing the posture classification task.

### 3.3. Generative AI and LLMs-Based Behavior Analysis

In the backend behavioral analysis component of this study, Generative AI services are integrated to enhance the accuracy of behavior pattern recognition and improve the system’s intelligent responsiveness. Although Large Language Models (LLMs) are not originally designed to process numerical sensor data—such as RSSI signals—and cannot directly interpret the time-series variations of raw RSSI data, their semantic understanding and inference capabilities can still be effectively utilized through proper data preprocessing and translation strategies [29].

In this study, a large volume of RSSI data collected from real-world environments is cleaned and filtered. The data is then labeled and semantically translated based on different measurement heights and device positions, transforming it into semantically structured textual descriptions, which are then provided as input to the LLM.

By training the model to understand the semantic relationships between various RSSI patterns and user postures, Generative AI can generate highly probable inference results for new semantic RSSI inputs. For example, when the model receives a description such as “DCU 1 RSSI is −78, DCU 2 is −81, DCU 3 is −74, similar to the known characteristics at 60 cm height,” the LLM returns a probability percentage indicating that the corresponding height is 60 cm, along with a textual explanation of the inferred behavior.

This approach not only deepens the model’s interpretation of the data but also enhances the system’s explainability and adaptability in complex scenarios—laying the groundwork for intelligent care alert and decision-making mechanisms in future applications.

### 3.4. Environmental Data Collection Method

**Figure 17:**
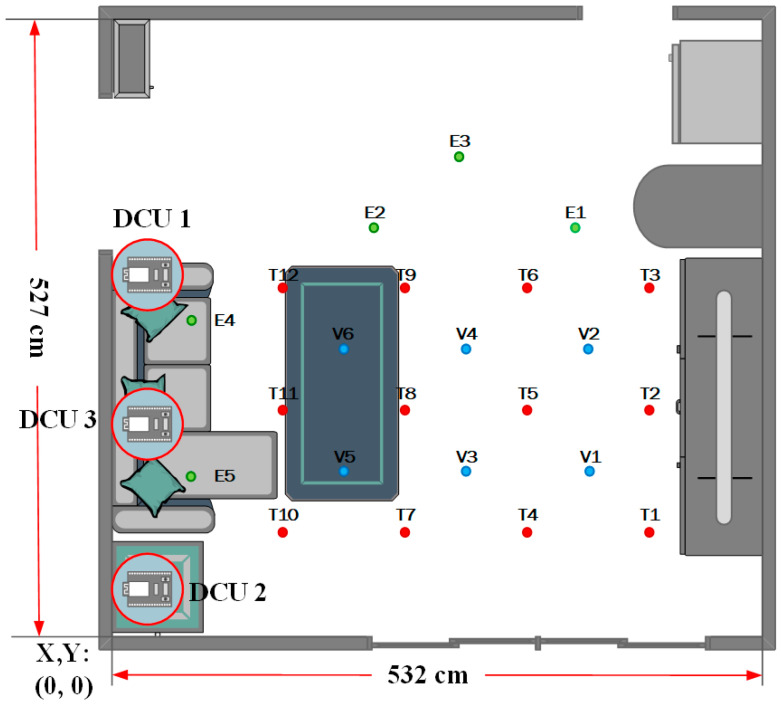
Environmental measurement coordinate configuration diagram. Red bullet means training points; Blue bullet means validation points; and Green bullet means evaluation points.

Environmental data generated by different behavior patterns provides crucial insights for subsequent data analysis and decision-making. Therefore, the selection of measurement locations is a critical factor in ensuring accurate and representative data collection. To capture realistic environmental data, this study selects the living room of a residential environment as the experimental measurement site. During the selection process for reference points, considerations include the placement of furniture and objects, as well as the potential movement range of the test subject, ensuring that the measurement results remain representative and reliable. As illustrated in Figure 17, the reference points are categorized into three main types:

1. Training Points (T-Series):
  - Used for training the behavior pattern analysis model.
  - Reference points are spaced 90 cm apart, starting from T01 to T12.
  - Each reference point collects data at 0 cm, 60 cm, and 90 cm heights to represent different behavior patterns.
2. Validation Points (V-Series):
  - Used to validate the accuracy of behavior pattern analysis.
  - Reference points are positioned between V01 and V12, starting from V01 and spaced 90 cm apart, extending to V06.
  - V01 to V04 collect data at 0 cm, 60 cm, and 90 cm heights.
  - V05 and V06 are located on a tabletop (65 cm height), so only 90 cm height data is collected.
3. Evaluation Points (E-Series):
  - Used to test behavior pattern analysis outside the predefined reference range.
  - Reference points are selected randomly within the living room rather than following a fixed interval.
  - To prevent excessive proximity, each E-point is spaced at least 60 cm apart.
  - E01 to E03 collect data at 0 cm, 60 cm, and 90 cm heights.
  - E04 and E05, positioned on sofa seats (approximately 50 cm height), collect data only at 60 cm and 90 cm heights.

At reference point T, the remaining space below is close to walls, glass doors, and storage cabinets, which makes this location prone to signal interference during measurement. Therefore, reference point E is primarily placed in the upper, more open area of the living room layout to reduce the impact of such interference.

### 3.5. Statistical Analysis and Experimental Design Description

To enhance the credibility and reproducibility of the experimental results, this study provides further details on statistical analysis and experimental design as follows:

1. Experimental Design:
  The behavior recognition experiment in this study was conducted with a single device, aiming to preliminarily verify the feasibility of the system. The smartwatch performed three actions—lying down, sitting, and standing—in a simulated living room environment, with RSSI signals measured at heights of 0 cm, 60 cm, and 90 cm. Each behavior was tested in 12 repeated trials, during which RSSI data were continuously collected for 10 min per trial.
  To avoid the influence of measurement order on the results, a randomized sequence was used for performing different actions. After data collection, 70% of the data was used to train the Decision Tree model, while the remaining 30% served as a test set for model validation.
2. Statistical Analysis:
  To assess the stability of the RSSI signals and the consistency of the model’s recognition performance, this study calculated the mean and standard deviation of RSSI values for each behavior pattern, with the results being displayed in tables and figures in the Results Section. Additionally, to comprehensively evaluate the classification performance of the Decision Tree model for each behavior pattern, indicators such as F1-score, Accuracy, and confusion matrix were calculated.
  The statistical results demonstrated that the RSSI distributions of each behavior pattern were clearly distinct, and the classification model consistently maintained an accuracy rate above 98% on the test set, indicating strong recognition capability and result stability of the proposed method.

## 4. Results

### 4.1. Environment Setup and RSSI Data Collection

**Figure 18:**
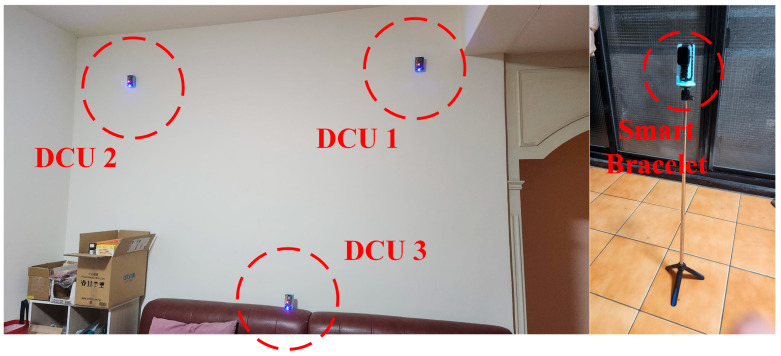
DCU device and smart wristband deployment implementation.

The placement of DCU devices was constrained by the testing environment. Among the four directions in the living room, one side consisted of glass doors, another side had a television, and another side was occupied by a bookshelf. As a result, the wall behind the sofa was the only viable installation area for the DCU devices. According to the data measurement setup, the DCUs were installed on the left-side wall, as shown in Figure 18. However, the placement of DCU 3 was restricted by the position of the sofa, preventing its height from being further lowered. On the right side of Figure 18, the scenario of a user wearing a smart wristband is simulated. Based on the environmental data collection setup, the wristband’s height was adjusted accordingly. The DCU devices were installed at designated positions within the living room to ensure data accuracy and measurement reliability.

To investigate the impact of different BLE RSSI measurement layouts on signal strength performance, this study improves upon the RSSI data collection methods used in previous research. Initially, three DCU devices were arranged in a horizontal layout near the ceiling. An alternative configuration involved installing only two devices on the ceiling, while the third device was placed at a lower position to simulate a vertical observational angle, thereby enabling the identification of spatial relationships between device height and the smartwatch.

**Figure 19:**
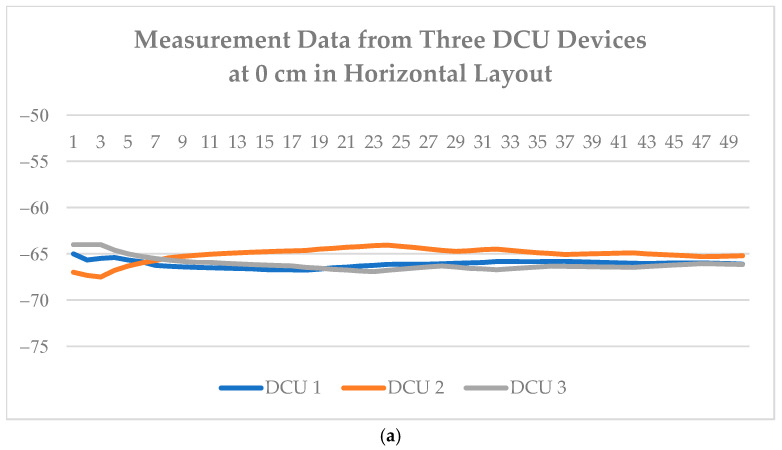

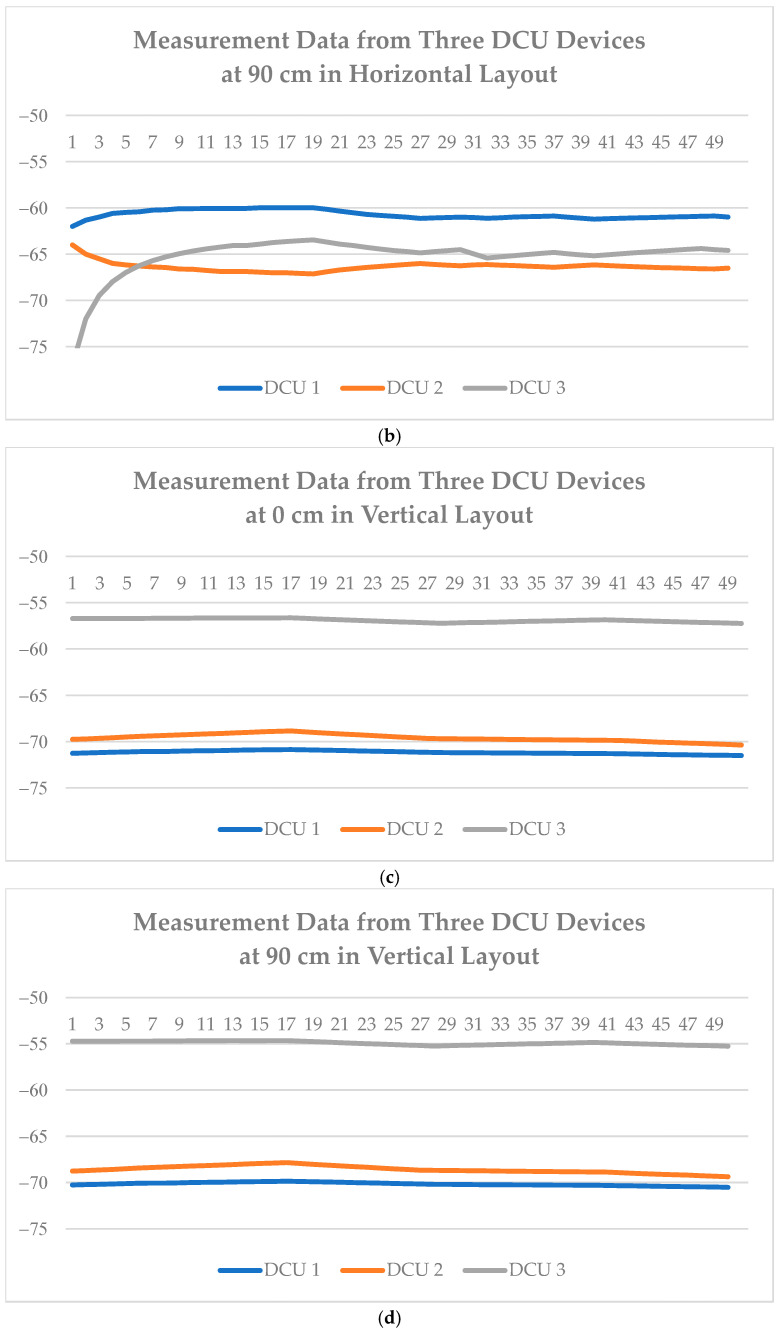
Data variations of DCU devices at different heights in various layouts: (**a**,**b**) represent the horizontal layouts; (**c**,**d**) represent the vertical layouts.

Figure 19 illustrates the RSSI signal collection results under both horizontal and vertical layouts at different heights. The results show that in the horizontal layout, regardless of whether the smartwatch is positioned at 0 cm or 90 cm, the variation trends in RSSI values across the three DCUs remain relatively stable. This indicates that when the device heights are consistent, RSSI is primarily influenced by horizontal obstructions and distance.

In contrast, the vertical layout more clearly reflects the impact of height differences on RSSI values. When the smartwatch is positioned at a height of 0 cm, DCU 3—installed at a lower level—records significantly stronger RSSI signals. However, as the smartwatch height increases to 90 cm, the RSSI detected by DCU 3 drops noticeably, and the differences between its values and those of DCU 1 and DCU 2 become smaller. This indicates that DCU 3 is more sensitive to changes in the smartwatch’s height.

On the other hand, DCU 1 and DCU 2 exhibit relatively minor RSSI variations across both height levels, suggesting that they are less responsive to changes in height. This may be attributed to their ceiling-mounted positions, where the distance to the smartwatch remains relatively large, and changes in vertical position result in only small relative shifts in spatial alignment.

Based on the above results, DCU 3 exhibits the most significant response to height changes in the vertical layout, indicating its critical role in height recognition and behavior inference. The vertical layout proves to be more effective in capturing relative height variations between the device and the user, making it well suited for non-visual behavior recognition and indoor care system design. In contrast, the horizontal layout is more appropriate for general planar positioning or area coverage detection scenarios.

**Figure 20:**
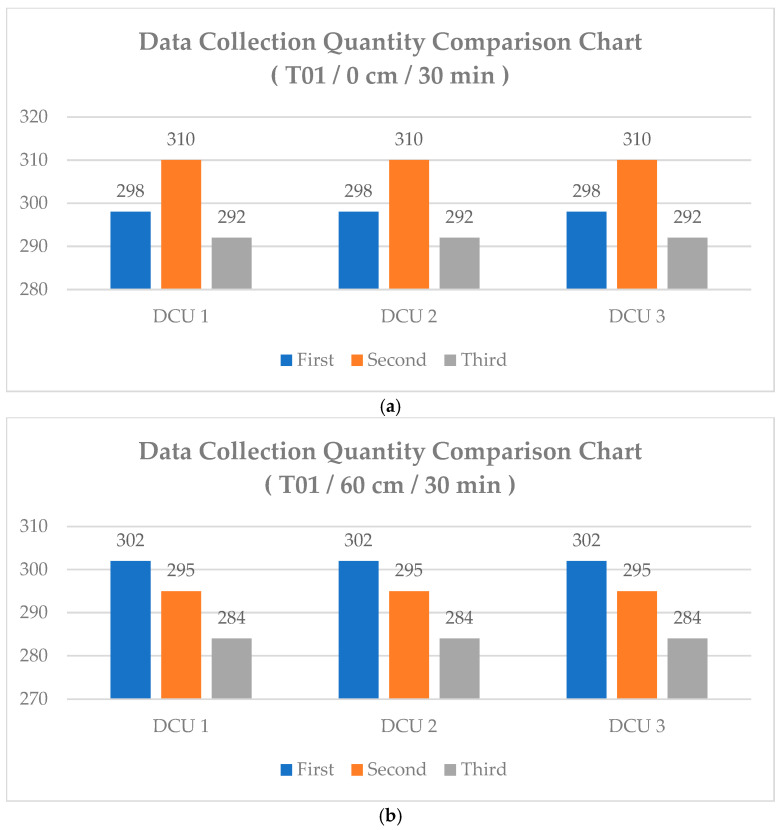

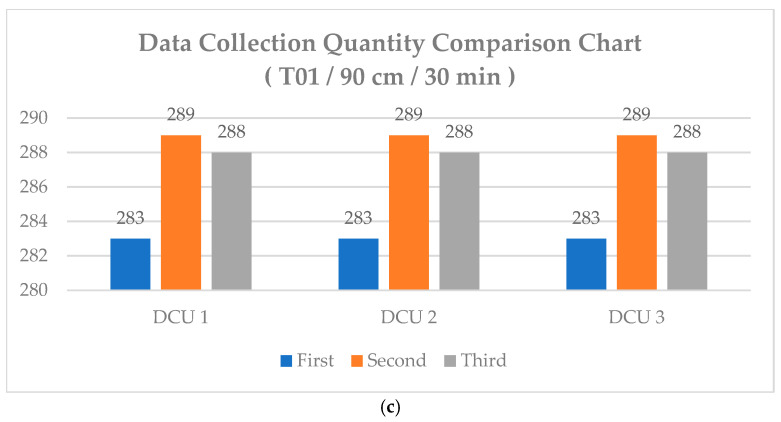
Comparison chart of data collection quantity at points T01: (**a**) 0 cm/30 min, (**b**) 60 cm/30 min, (**c**) 90 cm/30 min.

This study improved the data collection method by implementing a strategy where RSSI data is uploaded only after all three DCU devices have received signals. This approach effectively eliminates asynchronous data uploads among different DCU devices and reduces significant variations in data volume across DCUs. As illustrated in Figure 20, the distribution of collected data across all DCU devices is compared. The results show that, for the same location but different heights, the difference between the maximum and minimum number of data points does not exceed 20 records. Additionally, under conditions with minimal interference, each DCU was able to collect at least 250 data points, demonstrating the stability of the proposed data collection method. Due to its installation above the sofa, DCU 3 was more exposed to environmental interference sources than DCU 1 and DCU 2, resulting in a more noticeable signal reception delay. Under this mechanism, since data is stored only after all DCUs complete reception, the total RSSI data count is lower than when DCU 1 and DCU 2 independently detect and immediately upload data. For example, within a 30 min data collection period, DCU 1 and DCU 2 could each independently upload approximately 400 data points. However, under the synchronized collection approach, the total number of received data points was slightly reduced due to delays introduced by DCU 3.

### 4.2. Machine Learning-Based Behavior Classificaton

During the selection process for ML-based analysis tools, various methods were tested, and Decision Tree was ultimately chosen for behavior pattern analysis. Decision Tree performs well in handling nonlinear relationships within data. Compared with other linear prediction models, it offers better performance in classification and prediction tasks involving nonlinear data [30]. This study utilizes the PyCaret library for machine learning automation. PyCaret is designed to enhance the efficiency of machine learning processes by offering a variety of data preprocessing tools and performance visualization features. It includes a wide range of algorithms, including the commonly used Decision Tree. In the PyCaret setup, the categorical_features parameter is used to include the three distances from the DCUs and the timestamp as categorical features for training. Additionally, the number of folds for stratified k-fold cross-validation is set to five. Stratified k-fold is an advanced version of k-fold cross-validation that not only splits the dataset for validation but also ensures that the sample proportions in the training and test sets remain consistent with those in the original dataset.

During the data processing stage, in order to prevent the machine learning model’s generalization ability from being compromised due to high similarity between training and test data, this study first randomly shuffled and redistributed the dataset before dividing it into training and testing sets. This step helps avoid issues related to data leakage or biased evaluation, ensuring a more transparent and reliable process. As a result, the experimental outcomes may still include minor errors. Within PyCaret, the Feature Importance chart can be used to identify which features (columns) the model considers most influential in making predictions. This visualization provides valuable insights into the model’s decision-making process.

**Figure 21:**
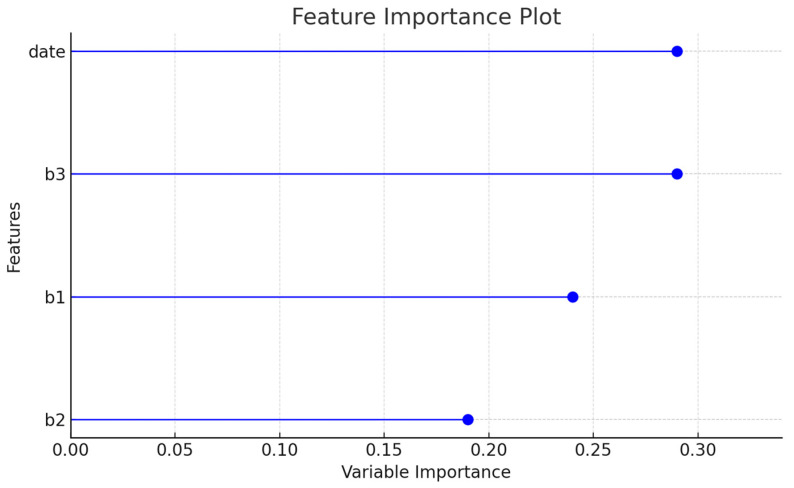
Feature importance of variables.

As illustrated in Figure 21, time variables and DCU 3 hold higher weights compared with DCU 1 and DCU 2. It is important to note that although DCU 1, DCU 2, and DCU 3 all serve as distance measurement references, their influence may vary due to environmental factors or human activities. In certain conditions, specific variables may have a more significant impact, leading the model to place greater emphasis on particular feature variables during analysis.

The weight of feature variables also influences the structure of the Decision Tree. When certain variables have a stronger impact, the Decision Tree may make classification decisions at higher-level leaf nodes, potentially ignoring lower-weighted variables. However, as shown in Figure 21, there are no extreme feature weight imbalances. This ensures that DCU 2 still maintains significant influence within the Decision Tree, retaining a reasonable decision weight in the classification process.

According to the data presented in Table 1, all evaluation metrics, including Accuracy, AUC (Area Under Curve), Precision, Recall, and F1-score, as well as more comprehensive error assessment metrics such as Cohen’s Kappa and Matthews Correlation Coefficient (MCC), achieved scores above 0.95. Given the minimal differences in these values, it can be inferred that the model demonstrates exceptionally high accuracy in data prediction while avoiding any bias toward specific data classes.

The consistent error rate and high evaluation scores indicate a strong compatibility between the characteristics of the dataset and the analysis method used in this study. Since the dataset includes numerical variables such as time and location, and Decision Tree is inherently capable of comparing numerical values in its leaf node splitting conditions, it proves to be a suitable model for this type of predictive task.

The prediction targets in this study encompass three height levels (0 cm, 60 cm, and 90 cm). This means that even if the model performs splitting at any internal node, it can generate at most three leaf nodes, thereby reducing the overall complexity of the model. Experimental results indicate a correlation between the low complexity of the training data and the high prediction accuracy. This suggests that human indoor behavior patterns exhibit a certain level of regularity, with minimal influence from outliers or unusual actions, leading to an exceptionally low error rate.

### 4.3. LLM-Based Behavior Prediction and Analysis

For the LLM analysis component, this study first processes and uploads the DCS-collected data from the test points, specifically from locations T01 to T12 at various heights, to be analyzed by the LLM. The data format used for this analysis is illustrated in Table 2.

To effectively utilize BLE RSSI data in Large Language Model (LLM) analysis, organizing the data in a tabular format, as illustrated in the figure, is essential. The column-based design of the table clearly separates each data entry by timestamp, the RSSI values received from the three receivers (DCU-1, DCU-2, DCU-3), and the corresponding behavior label. This structure enhances the semantic and structural consistency of the data, enabling the LLM to better understand and infer meaningful relationships during analysis.

For the LLMs-based analysis, the study first provided data from testing points P01 to P12 to the LLMs model for evaluation. The LLMs identified distinct statistical characteristics associated with RSSI values at different heights and further calculated the mean RSSI values and standard deviations for each height, as shown in Table 3 and Table 4. In Table 4, when the height is 0 cm, DCU 3 exhibits greater signal fluctuation. This is primarily because DCU 3 is positioned above the backrest of the sofa, and when the smart wristband is placed on the ground, the signal transmission path is more likely to be obstructed by the living room table and objects on its surface, leading to higher signal deviation. As the measurement height increases to 60 cm, interference factors affecting the smart wristband’s signal are reduced, and DCU 3’s standard deviation decreases, indicating that RSSI fluctuations are more stable. At 90 cm, obstructions between DCU 3 and the smart wristband are minimized, causing its standard deviation to align more closely with those of other DCUs. As a result, the overall data stability improves.

After establishing a basis for height classification, this study further tested coordinate data not included in the training dataset to evaluate the applicability of LLMs in height analysis. The T01 testing point was selected for height prediction analysis. LLMs used Euclidean distance to compute the distance between the given coordinate data and known height data points. Based on these distances, the model assessed the probability distribution across different height levels [31]. As shown in Table 5, the LLMs analysis results indicate that the probability of V01 being at 0 cm is 42.26%, which is higher than the probabilities for other heights, successfully identifying its actual height.

To prevent Generative AI from producing incorrect analysis results, this study independently executed computations based on the analysis methods and code suggested by the LLMs and cross-verified the results with the data generated by the LLMs. After confirming the accuracy of the calculations, coordinate data from different measurement points and heights were sequentially provided to the LLMs for analysis. As shown in Table 6, during the 60 cm height data test, the LLMs successfully identified the actual height position represented by the data.

Additionally, since reference points V05 and V06 are located on the living room table, which has a height of approximately 65 cm, data collection at lower heights (0 cm and 60 cm) was not feasible. Therefore, V05 and V06 were only included in the 90 cm height test. As shown in Table 7, when the test height was 90 cm, some coordinates (V04 and V05) were affected by environmental interference, leading to incorrect height classification. However, overall, the accuracy of behavior pattern analysis for the V-series test points reached 85.71%, demonstrating the stability and reliability of LLMs in height estimation.

In this study, two different types of reference points were set up for comparison and analysis. One type of reference point was positioned within the training data range, primarily used to evaluate LLM’s height estimation accuracy in a known environment. The other type was placed outside the training data range to test LLM’s adaptability in an unknown environment.

Reference points V01 to V06, located within the training data range, correspond to the T01 to T12 measurement area and are used to validate LLM’s accuracy in a familiar data environment. In contrast, reference points E01 to E05 are positioned outside the T01 to T12 range, allowing for an analysis of how environmental variations affect LLM’s prediction results.

As shown in Table 8, Table 9 and Table 10, LLMs analyzed the BLE device height percentages and prediction accuracy for reference points E01 to E03 at different heights. Since E01 to E03 are outside the environmental dataset range, there is less reference data available, leading to lower accuracy in LLM’s behavior pattern analysis. Compared with the V01 to V06 analysis results, the overall accuracy for the E-series reference points was only 33.33%, indicating that LLM’s height estimation capability is significantly affected in unknown environments.

The DCU circuit board utilizes a PCB antenna design, which is affected by directional limitations in signal transmission and reception. Reference points E04 and E05 were located too close to the dead zones of the DCU devices, resulting in a signal angle of less than 15 degrees between the smart wristband and the DCUs, which significantly impacted signal stability.

At reference point E04, DCU 1 was almost unable to receive valid data, while DCU 2, positioned near a corner, experienced a signal reflection effect. Although DCU 2 could still receive data, the number of received signals was limited to approximately 100 records per 30 min. In comparison, DCU 3—being positioned closer to the smart wristband—is less affected by signal reception angles and receives a relatively larger volume of data, approximately 160 entries per 30 min. For reference points E04 and E05, when using the approach of “uploading only after receiving data from all DCUs,” the actual number of RSSI entries received in the database was fewer than 20 per 30 min. Additionally, the time intervals between entries were inconsistent and excessively large, making it impossible to construct a valid time-series.

Due to the severe insufficiency of RSSI data collected at these two reference points, there were not enough samples for effective statistical or model analysis. As a result, reference points E04 and E05 were ultimately excluded from this analysis.

## 5. Conclusions

The results of this study demonstrate that BLE DCUs can successfully collect BLE signals from smart wristbands and, through ML or LLMs-based behavior analysis, accurately determine the device height corresponding to different behavior patterns. For ML analysis, Decision Tree was used as the classification model. To simulate real-time monitoring conditions, the study directly analyzed raw, unfiltered data without applying smoothing or optimization. The results indicate that Decision Tree effectively identifies behavior patterns based on RSSI data. Even when reference points were located outside the environmental dataset range, the model was still able to correctly predict device height.

For LLMs analysis, data must first undergo smoothing and optimization processing; otherwise, using raw data directly can lead to inaccurate predictions. During the AI training process, continuous adjustments and guidance are necessary to ensure the model identifies the most suitable data analysis methods. Additionally, since Generative AI is prone to hallucination effects (Hallucination), multi-source verification is required to validate data accuracy and refine results, thereby reducing incorrect inferences during the early training stages.

This study proposes using ESP32 as a BLE DCU device to collect RSSI data. However, due to the built-in PCB antenna design of the ESP32, signal reception is highly susceptible to environmental interference, and the directionality of the antenna may affect measurement accuracy. For future research, adopting an external omnidirectional antenna could be considered to enhance noise resistance, ensuring greater stability and accuracy in data collection.

## Figures and Tables

**Table 1:** Model’s mean error and prediction rate.

Model	Accuracy	AUC	Recall	Prec.	F1	Kappa	MCC
Decision Tree Classifier	0.98	0.9851	0.98	0.98	0.98	0.9698	0.9698

**Table 2:** Data from reference point T01 at 0 cm, used for LLM analysis.

No.	Timestamp	DCU 1	DCU 2	DCU 3	Label
1	4 December 2024 01:48	−73.49999146	−72.83333049	−62	Fall
2	4 December 2024 01:48	−73.28570107	−72.85714107	−61.85713893	Fall
3	4 December 2024 01:48	−73.12498211	−71.87495977	−63.12504648	Fall

**Table 3:** Statistical characteristics of average RSSI at different heights.

Model	DCU 1	DCU 2	DCU 3
0 cm	≈−64.4	≈−63.9	≈−65.7
60 cm	≈−64.9	≈−65.1	≈−65.0
90 cm	≈−68.7	≈−68.1	≈−59.3

**Table 4:** Statistical characteristics of RSSI standard deviation at different heights.

Model	DCU 1	DCU 2	DCU 3
0 cm	≈5.8	≈5.7	≈8.0
60 cm	≈5.8	≈4.4	≈6.0
90 cm	≈5.2	≈3.8	≈5.3

**Table 5:** Percentage and accuracy of BLE device height estimation by LLMS for points V01 to V04 at 0 cm. Bold values indicate the highest percentage in each row (i.e., the most likely location identified for each height).

Model	V01_Z0	V02_Z0	V03_Z0	V04_Z0
0 cm	**42.26%**	**41.03%**	**44.98%**	**43.37%**
60 cm	36.90%	37.80%	37.49%	41.06%
90 cm	20.84%	21.17%	17.53%	15.58%
Current	1	1	1	1

**Table 6:** Percentage and accuracy of BLE device height estimation by LLMS for points V01 to V04 at 60 cm. Bold values indicate the highest percentage in each row.

Model	V01_Z60	V02_Z60	V03_Z60	V04_Z60
0 cm	30.84%	28.45%	32.00%	36.11%
60 cm	**37.47%**	**38.31%**	**47.42%**	**38.58%**
90 cm	31.69%	33.24%	20.58%	25.31%
Current	1	1	1	1

**Table 7:** Percentage and accuracy of BLE device height estimation by LLMS for points V01 to V06 at 90 cm. Bold values indicate the highest percentage in each row.

Model	V01_Z90	V02_Z90	V03_Z90	V04_Z90	V05_Z90	V06_Z90
0 cm	17.20%	29.65%	28.49%	31.28%	34.85%	26.04%
60 cm	20.53%	31.18%	30.87%	**37.68%**	**34.76%**	28.33%
90 cm	**62.26%**	**39.17%**	**40.63%**	30.51%	30.39%	**45.63%**
Current	1	1	1	0	0	1

**Table 8:** Percentage and accuracy of BLE device height estimation by LLMS for points E01 to E03 at 0 cm. Bold values indicate the highest percentage in each row.

Model	E01_Z0	E02_Z0	E03_Z0
0 cm	**38.89%**	35.42%	38.05%
60 cm	37.50%	**36.70%**	**39.26%**
90 cm	23.61%	27.88%	22.69%
Current	1	0	0

**Table 9:** Percentage and accuracy of BLE device height estimation by LLMS for points E01 to E03 at 60 cm. Bold values indicate the highest percentage in each row.

Model	E01_Z60	E02_Z60	E03_Z60
0 cm	**43.06%**	31.63%	**38.44%**
60 cm	40.60%	**44.80%**	37.10%
90 cm	16.34%	23.57%	24.46%
Current	0	1	0

**Table 10:** Percentage and accuracy of BLE device height estimation by LLMS for points E01 to E03 at 90 cm. Bold values indicate the highest percentage in each row.

Model	E01_Z90	E02_Z90	E03_Z90
0 cm	29.50%	28.93%	36.33%
60 cm	**55.71%**	32.29%	**39.36%**
90 cm	14.08%	**38.78%**	24.31%
Current	0	1	0

## Data Availability

Data are contained within the article.
